# The role of innate immune cells in the colorectal cancer tumor microenvironment and advances in anti-tumor therapy research

**DOI:** 10.3389/fimmu.2024.1407449

**Published:** 2024-07-19

**Authors:** Wenxuan Liu, Tianrui Kuang, Li Liu, Wenhong Deng

**Affiliations:** Department of General Surgery, Renmin Hospital of Wuhan University, Wuhan, Hubei, China

**Keywords:** innate immune cells, colorectal cancer, tumor microenvironment, immunotherapy, immune cell

## Abstract

Innate immune cells in the colorectal cancer microenvironment mainly include macrophages, neutrophils, natural killer cells, dendritic cells and bone marrow-derived suppressor cells. They play a pivotal role in tumor initiation and progression through the secretion of diverse cytokines, chemokines, and other factors that govern these processes. Colorectal cancer is a common malignancy of the gastrointestinal tract, and understanding the role of innate immune cells in the microenvironment of CRC may help to improve therapeutic approaches to CRC and increase the good prognosis. In this review, we comprehensively explore the pivotal role of innate immune cells in the initiation and progression of colorectal cancer (CRC), alongside an extensive evaluation of the current landscape of innate immune cell-based immunotherapies, thereby offering valuable insights for future research strategies and clinical trials.

## 1 Introduction

**Figure 1:**
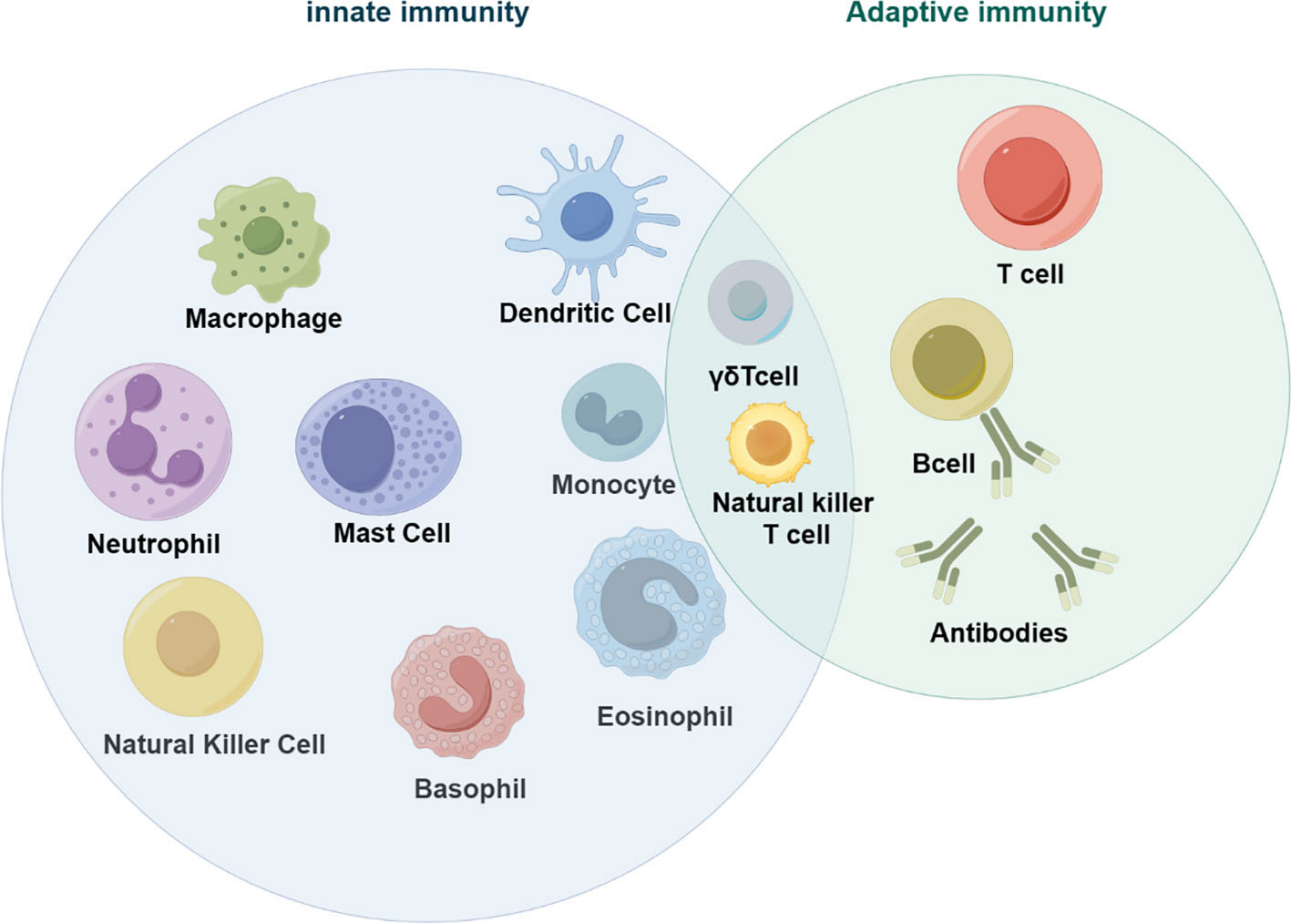
Innate immune cells in tumors (by figdraw).

Colorectal cancer (CRC) is a global disease that poses a serious threat to human life and health, and is a common malignant tumor of the gastrointestinal tract. According to the latest cancer statistics, colorectal cancer has the third highest incidence rate globally and the second highest mortality rate after lung cancer (1). The tumor microenvironment refers to the complex surroundings in which tumor cells exist and grow. This includes the surrounding blood vessels, immune cells, fibroblasts, myeloid-derived inflammatory cells, a variety of signaling molecules, and the extracellular matrix (2). These cellular interactions facilitate immune evasion by tumor cells, thereby serving as a cytological mechanism underlying tumor progression and metastasis (3). There is increasing evidence that both innate and adaptive immune cells play a crucial role in the tumor microenvironment (TME) (**Figure 1**). These cellular components, encompassing macrophages, neutrophils, natural killer cells, dendritic cells, bone marrow-derived suppressor cells, T lymphocytes, and B lymphocytes, actively participate in tumorigenesis and the progression of tumors. In particular, innate immune cells secrete cytokines, chemokines, growth factors, and extracellular matrix (ECM) proteins through a complex communication network. These are regulated by interactions with colorectal cancer cells and affect the survival and progression of colorectal cancer. In addition to identifying the molecular events occurring in tumor cells, it is important to have a comprehensive understanding of the pathogenic mechanisms of CRC. This includes defects in the tumor microenvironment (TME), such as immune cell infiltration, immune defense, immune surveillance and immune homeostasis. It is crucial to not overlook these factors (3). This review focuses on innate immune cells in the tumor microenvironment of colorectal cancer. It also discusses the immune mechanisms that promote tumor progression. The aim is to provide ideas for finding new therapeutic approaches for CRC patients.

## 2 Innate immune cells in the colorectal cancer microenvironment

### 2.1 Macrophage

Macrophages are an important component of the tumor microenvironment and play an important role in maintaining the innate immune response, tissue homeostasis and inflammation. Macrophages are recruited around tumors to form tumor-associated macrophages (TAM), which can be induced into M1 and M2 types (4). M1 macrophages are also known as pro-inflammatory macrophages due to their secretion of large amounts of pro-inflammatory cytokines, including IL-1β and tumor necrosis factor-α (TNF-α). M1 macrophages are primarily induced by lipopolysaccharide (LPS) and interferon-γ (IFN-γ). In the presence of these substances, macrophages undergo a process of polarization, acquiring the M1 phenotype. M2 macrophages are known as anti-inflammatory macrophages due to their production of mainly anti-inflammatory factors, including IL-10, transforming growth factor-β (TGF-β), and arginase 1 (Arg1). M2 macrophages can be activated by interleukin (IL)-4 and IL-13.During carcinogenesis, macrophages initially exhibit an M1-like polarization with anti-tumor properties, leading to enhanced elimination of tumor cells. However, as the tumor progresses, macrophages undergo a shift towards an M2-like polarization state that promotes tumor progression. Studies have demonstrated that well-differentiated tumor-associated macrophages (TAMs) are associated with unfavorable prognosis and reduced overall survival (5).

### 2.2 Neutrophil

Neutrophils are the most abundant white blood cells in the human circulation. During carcinogenesis, neutrophils are transformed into tumor-associated neutrophils (TAN) after migration to tumor tissue. TGF-β in the tumor microenvironment induces their differentiation into either N1 or N2 types (6). Neutrophils have cancer-inhibiting (7) and cancer-promoting effects. Tumor growth, proliferation, and aggressiveness are increased by neutrophil depletion.

### 2.3 Natural killer cell

Natural killer cells (NK cells) are immune cells derived from bone marrow lymphoid cells that play a crucial role in anti-tumor immunity and immunomodulation. The effectiveness of NK cells in attacking tumors depends on the receptors they express. These receptors are classified as either activating or inhibitory, and they regulate the activation of NK cells. Activated NK cells can kill tumor cells through two mechanisms: natural killer toxicity and the ADCC pathway.

### 2.4 Dendritic cells

Dendritic cells are a type of antigen-presenting cell in the immune system that link the adaptive and innate immune systems to initiate and maintain T-cell-mediated immunity against tumors. They have a close relationship with tumorigenesis and progression, and a high number of DCs infiltrating most solid tumors is associated with a good prognosis (8).

### 2.5 Myeloid-derived suppressor cells

Myeloid derived suppressor cells have strong immunosuppressive activity. They are implicated in the regulation of immune responses under many pathological conditions and are strongly associated with poor clinical outcomes in cancer (9). Myeloid derived suppressor cells classified according to their origin as granulocyte/polymorphonuclear cell MDSCs (PMN-MDSCs) and monocyte MDSCs (M-MDSCs). Their main characteristic is the ability to suppress the immune response.

## 3 Functional mechanisms of innate immune cells in CRC

### 3.1 Tumor-associated macrophages

Macrophages can promote or suppress tumor immunity by polarizing into different types of tumors and play a key role in the tumor microenvironment. They produce various molecules that interact with other immune cells and tumor cells to further influence CRC progression (4). CRC cells secrete extracellular vesicles EV, and miRNAs carried by extracellular vesicles induce macrophage polarization, which plays an important role in regulating colorectal cancer cell migration and invasion (10). For instance, miR-195-5 regulates the NOTCH pathway, which affects interleukin 4 (IL-4)-associated M2-like TAM polarization in colorectal cancer (11). Other miRNAs also play an important role. The exosome circVCP derived from CRC cells regulates the miR-9-5p/NRP1 axis, promoting cell proliferation, migration, and invasion. Additionally, it induces macrophage M2 polarization and inhibits macrophage M1 polarization, thereby facilitating the progression of CRC (12). M2 macrophage-derived extracellular vesicle-mediated miR-186-5p targets DLC1 to promote colon cancer progression (13).

TAM induces epithelial-mesenchymal transition (EMT) through various pathways to enhance colorectal cancer migratory invasion. The EMT program is induced by TAM to enhance CRC migratory invasion by regulating the JAK2/STAT3/miR-506-3p/FoxQ1 axis. This leads to the production of chemokine 2 (CCL2), which promotes macrophage recruitment (14). Platelets promote tumor metastasis. Tumor cells interact with platelets to generate chimeric extracellular vesicles. These vesicles inhibit primary tumor growth through the activation of tumor-destroying macrophages, while promoting tumor metastasis through EMT and endothelial activation (15). The Metastasis-associated regenerative liver 3 protein phosphatase (PRL-3) promotes invasion and metastasis of colorectal cancer cells by activating the epithelial-mesenchymal transition through the MAPK pathway in TAMs. Additionally, PRL-3 promotes angiogenesis in CRC cells through activation of the NF-κB pathway (16). The SNAIL protein facilitates the transformation of epithelial tumors. Additionally, transformed mesenchymal stromal cells secrete CXCL2, which promotes the infiltration of M2 macrophages and the metastasis of tumor cells (17).

The secretion of chemokines can induce the polarization of M2-type macrophages. Most studies have focused on the Wnt5a/CaMKII/ERK/CCL2 axis. Additionally, HMGA2 directly binds to the STAT3 promoter, activating its transcription and subsequently inducing CCL2 secretion, which promotes macrophage recruitment (18). Wnt5a induces M2 polarization of TAMs by regulating interleukin 10 (IL-10) secretion through the CaMKII-ERK1/2-STAT3 pathway. This ultimately promotes tumor growth and metastasis in colorectal cancer (19). PIPkIγ promotes the activation of the PI3K-Akt-mTOR signaling pathway, leading to an increase in STAT3 phosphorylation levels. This, in turn, triggers CCL2 transcription, enhancing the recruitment of tumor-associated macrophages (20).The tissue inhibitory factor of matrix metalloproteinases 1 (TIMP1) promotes macrophage migration and participates in macrophage M2 polarization through the activation of ERK1/2/CLAM1 and CCL2 (21). The CCL2-CCR2 signaling pathway is involved in the polarization of M2-type macrophages. TCF4 enhances liver metastasis in colorectal cancer by regulating tumor-associated macrophages through CCL2/CCR2 signaling (22). Colorectal cancer growth is also promoted by the overexpression of transforming growth factor beta (TGF-β) in tumor-associated macrophages (23, 24). The secretion of chemokine 11 (CXCL11) increases and enhances the expression of transforming growth factor in tumor-associated macrophages, which in turn promotes the metastasis of colorectal cancer cells (25). The chemokines interleukins 4, 6, 8, and 10 promote the development of colorectal cancer by converting macrophages to tumor macrophage M2 type (11, 26–28). Other signaling pathways NK-κB (29, 30), I3K/AKT (31, 32), NOTCH (11) and tumor-associated macrophage M2 are involved in CRC development.

*In vivo*, certain proteins display tumorigenic activity and advance CRC progression by inducing M2 polarization of TAM. CRC cells activate STAT6 and the transcription factor KLF via the malignant fibrous histiocytoma amplicon sequence 1 (MFHAS1), which has tumorigenic activity, and induce M2 polarization of TAM, promoting CRC progression (12, 33, 34). Intercellular adhesion molecule-1 (ICAM-1) is a transmembrane glycoprotein that belongs to the immunoglobulin superfamily. It mediates cell-to-cell interactions and intra- and extracellular signaling during the immune response. ICAM-1 down-regulates cellular proliferation in the tumor microenvironment, inhibiting M2 macrophage polarization and metastatic tumor progression (35). Tumor-associated macrophages promote cancer cell migration and invasion by inducing serum amyloid A1 (SAA1) at the early colorectal cancer (CRC) invasion front (36). Immunoglobulin-like transcript 5 (ILT5) functions as a negative regulator of myeloid cell activation and directs M2-like polarization of tumor-associated macrophages (37).

Tumor-associated macrophages (TAMs) can inhibit the anti-tumor activity of T cells. Our research has shown that deletion of RNA N6-adenosylmethyltransferase Mettl14 in TAMs promotes CD8 T cell dysfunction and tumor growth (38). miR-21-5p and miR-200a work together to induce M2-like polarization of macrophages and increase PD-L1 expression by regulating the PTEN/AKT and SCOS1/STAT1 pathways. This leads to a decrease in CD8T cell activity and an increase in tumor growth (34). Additionally, Chemokine 8 (CXCL8) induces M2 macrophage polarization and inhibits CD8T cell infiltration, creating an immunosuppressive microenvironment in colorectal cancer (39). The p65/STAT3-CSN5-PD-L1 pathway is used by colorectal cancer cells to promote immune escape, with the assistance of macrophage-derived chemokine 5 (CCL5) (40).

Although macrophages in colorectal cancer (CRC) tend to convert to the M2 phenotype and act as tumor promoters, they can sometimes convert to the M1 phenotype and act as anti-tumor promoters. The protein Elastin microfibril interface 2 (EMILIN-2) triggers the activation of the Toll-like receptor 4/MyD88/NF-κB pathway and contributes to macrophage polarization towards the M1 phenotype (41). The axis between 1-acylglycerol-3-phosphate O-acyltransferase 4 (Agpat4) and lysophosphatidic acid (LPA) in CRC cells modulates p38/p65 signaling-dependent macrophage M1 polarization, promotes T-cell activation, and inhibits CRC progression (42). The extracellular matrix glycoprotein, spondin 2 (SPON2), derived from tumor cells, can inhibit tumor growth and invasion by indirectly inducing M1 poles. This is achieved through the up-regulation of cytokines expressed by IL2, CCL10, and colony-stimulating factor 2 (CSF2) in tumor cells (43). Mucin promotes the enrichment of M1-like macrophages both *in vivo* and *in vitro*. This process is dependent on the NOD-like receptor thermoprotein structural domain protein 3 (NLRP3). The absence of NLRP3 in macrophages attenuates the tumor suppressive effect of mucinophages (44). ILC1 promotes M1-like macrophage activation and inhibits colon cancer progression by secreting gamma interferon (IFN-γ) (45).

### 3.2 Neutrophil

Chronic inflammation contributes to the progression of colorectal cancer. The development and progression of colorectal cancer (CRC) is closely linked to the interaction between the inflammatory factors interleukins (ILs) and neutrophils (46). The overexpression of IL-17 is linked to a poor prognosis in colorectal cancer, whereas the production of IL-22 is associated with a favorable clinical outcome (47, 48). IL-22 is produced by CD4 and CD8 multifunctional T cells, which also produce IL-17 and IFN-γ (48). The blockade of IL-17 has been demonstrated to result in a reduction in the recruitment of neutrophils to the intestinal mucosa, as well as a reduction in the expression and production of inflammatory cytokines and the expression of the transcription factor STAT3 in the intestinal mucosa (49). Furthermore, the combination of drugs targeting the IL-17 signaling pathway with T-cell checkpoint inhibitors has the potential to enhance cancer immunotherapy (50). CXCL8 is a potent chemokine that attracts neutrophils, myeloid-derived suppressor cells (MDSC) and monocytes (46). Apoptotic cancer cells release CXCL8, which attracts neutrophils into the tumor, interacts with macrophages and regulates the immune microenvironment of CRC.IL-8 regulates tumor stem cells (CSC) in colon and lung cancer cells via glucose transporter protein 3 (GLUT3) and glucosamine fructose-6-phosphate aminotransferase (GFAT) induced glucoramidic (O-GlcNAc) modification Sample Properties (51). The superoxide dismutase (SOD2)-CXCL8-neutrophil recruitment axis may be a potential factor in the progression of colorectal cancer (52). The presence of low non-red lineage protein (SPTAN1) in colorectal cancer (CRC) cells has been shown to result in the release of higher levels of IL-8 and the subsequent migration of neutrophils, leading to an increased infiltration of immune cells (53). The development of colon tumors is promoted by epithelial hypoxia-inducible factor 2 alpha, which recruits neutrophils through the CXCL1-CXCR2 signaling axis (54). SMAD4 is a crucial transcription factor in TGFβ signaling and functions as a tumor suppressor in colorectal cancer. SMAD4 deficiency promotes the progression of colorectal cancer by attracting tumor-associated neutrophils through the CXCL1/8-CXCR2 axis (55). Exosomal RNA derived from tumor stem-like cells stimulates neutrophils to promote colon cancer tumorigenesis by secreting CXCL1 and CXCL2. These chemokines recruit neutrophils to promote tumorigenesis in colorectal cancer cells via IL-1β (56). The inflammatory mediator prostaglandin E2 (PGE2) specifically promotes colorectal cancer formation through its E2-EP2 signaling pathway (57). Prostaglandin receptor 2 (EP2) has been reported to promote colon tumorigenesis by amplifying inflammation and shaping the tumor microenvironment in neutrophils and tumor-associated fibroblasts (58). In TME of CRC, tumor-associated neutrophils (TAN) secrete anterior gradient protein 2 (AGR2) to promote migration of CRC cells (59). Neutrophils promote the growth of liver metastases from colorectal cancer in mice through fibroblast growth factor 2 (FGF2)-dependent angiogenesis (60). Neutrophils create neutrophil extracellular traps (NETs) after necrosis. These NETs are important for directly promoting cancer growth (61). Clostridium nucleatum (Fn)-induced NETs promote tumor metastasis through the Toll-like receptor 4 (TLR4)-reactive oxygen species (ROS) signaling pathway and the NOD-like receptor (NOD1/2)-dependent signaling pathway (62). Defective aryl hydrocarbon receptor nuclear translocator (ARNT) induces neutrophil extracellular traps (NET) and promotes colorectal development (63). Neutrophil extracellular traps (NETs) promote T cell depletion in the tumor microenvironment (61). It has beenIsekai Cheat Magician reported that polymorphonuclear neutrophils (PMN) induce a homologous recombination-deficient colorectal cancer (CRC) phenotype (64). Defects in MIR4435-2HG, a long non-coding RNA that suppresses tumors, increase the number of polymorphonuclear myeloid-derived suppressor cells (PMN-MDSCs) that infiltrate tumors and enhance their immunosuppressive potential, promoting the growth of colorectal cancer (65). CCL2 enhances the number and function of PMN-MDSCs, promoting colorectal cancer (66).

Neutrophils not only secrete relevant molecules but also promote cancer development and interact with other immune cells to influence tumor development. They inhibit colon cancer tumor-infiltrating T cells through matrix metalloproteinase (MMP)-mediated activation of transforming factor β (TGFβ) (67). Some evidence suggests that interactions between macrophages and CRC cells lead to neutrophil recruitment and that SAA1 secreted by CRC cells activates neutrophils to promote invasion (68). Neutrophil extracellular traps (NETs) play a crucial role in promoting cancer growth by inhibiting T-cell responses through metabolic and functional depletion (61). Several studies have shown that tumor-associated neutrophils (TAN) promote the spread of cancer cells to distant organs. TAN promote tumor invasion and angiogenesis, as well as migration, through the production of matrix metalloproteinase-9 (MMP-9), vascular endothelial growth factor (VEGF), and hepatocyte growth factor (HGF) at both primary and metastatic sites (69).

In some cases, Neutrophils may also contribute to cancer suppression by inhibiting tumor growth through miR-155-dependent down-regulation of RAD51 (64).

### 3.3 Natural killer cell

Natural killer cells are rare in the tissues of colorectal cancer patients (70). Although at low levels, in colorectal cancer TME, activated NK cells can exert a killing effect on tumors, mainly through two mechanisms: direct killing action and the ADCC (antibody-dependent cytotoxic effect) pathway. Tissue protease binds to the cell membrane and exhibits ADCC potential. Colorectal cancer tumor cells express histone S on the surface, and the binding of Fsn0503h to the surface-associated histone S leads to the targeting of NK cells to tumor killing (71). The effect of NK cells on tumors is mainly determined by the receptors expressed on their surface. TIGIT is an inhibitory receptor found on all human NK cells and a wide range of T cells. The Fap2 protein of Fusarium nuclei directly interacts with TIGIT, resulting in the inhibition of NK cytotoxicity (72).

NK cells secrete chemokines and cytokines to exert immunomodulatory functions. Studies have shown that interleukin-15 (IL-15) promotes the growth and activation of cytotoxic CD8T and NK cells (73). Significantly increased interleukin-21 expression in NK cells in the colorectal cancer microenvironment has an increased survival time for CRC patients (74). Mesenchymal stem cells (mSCs) specifically target T/NK cells to chemokine ligand 9 (CXCL9), leading to local anti-tumor immunity (75). NLRP3 inflammatory vesicles inhibit metastatic growth of colorectal cancer in the liver by affecting hepatic NK cell maturation through the interleukin-18 pathway and surface expression of the death ligand FasL (76). Black raspberry enhances the expression of the transcriptional effector Smad4 in colonic epithelial cells and natural killer cells, thereby inhibiting colorectal cancer (77). Lactic acid induces apoptosis in NK cells by reducing intracellular pH, leading to mitochondrial dysfunction. Colorectal cancer liver metastases (CRLM) tumors produce lactic acid, which lowers the pH of the tumor microenvironment, inducing apoptosis (78). The combined treatment of MC38 mouse colon cancer with methotrexate nanocouplings and dendritic cells with down-regulated IL-10R expression has been shown to modulate the tumor microenvironment and enhance the systemic anti-tumor immune response (79).

In colorectal cancer, a study has shown that cancer-associated fibroblasts enhance the enrichment of tumor-associated macrophages and inhibit the function of NK cells, which are associated with other cells in the tumor microenvironment (80). The anti-tumor effects of activated NK cells and anti-heat shock protein 70 (Hsp70) CAR-T cells are comparable (81). Macrophage STING signaling promotes NK cell inhibition of colorectal cancer liver metastasis via 4-1BBL/4-1BB co-stimulation (82). Targeting epidermal growth factor receptor (EGFR) counteracts inhibition of natural killer cell function by colorectal tumor-associated fibroblasts (83). 肿Crosstalk between NK cells and CD8T cells in the tumor microenvironment benefits the prognosis of colorectal cancer patients (84).

### 3.4 Dendritic cell

Dendritic cells (DCs) play an important role in the colorectal cancer tumor microenvironment. It has been shown that a high density of DCs at the dermal site contributes to tumor antigen trapping and that local inflammation induces DC maturation and migration into draining lymph nodes (85), thereby participating in tumor progression. In DC, mixed-spectrum kinase structural domain-like protein (MLKL) attenuates colonic inflammation and colitis tumorigenesis by inhibiting the inflammatory response through activation of the MEK/ERK pathway (86). Vascular endothelial growth factor (VEGF) affects the tumor microenvironment by inhibiting dendritic cell (DC) differentiation and promoting the accumulation of myeloid-derived suppressor cells. Additionally, heterodimeric interleukin-15 (hetIL-15) induces the intratumoral accumulation of CD8+ T and natural killer (NK) cells, which helps to control tumor growth (73). Dendritic cells from various sources, along with associated cytokines, contribute to tumor progression. Dendritic cells, specifically tumor-associated dendritic cells (TADCs), that surround colorectal cancer express recombinant human chemokine ligand 5 (CCL5), which promotes the migration and invasion of colorectal cancer cells (87). Conventional type 1 dendritic cells (cDC1s) are a widely studied subpopulation of DCs that play a central role in anti-tumor immunity. Their presence in the tumor microenvironment has been associated with improved prognosis in cancer patients. cDC1s contain the dead cell sensing receptor (DNGR-1), which inhibits tumor-infiltrating type I conventional dendritic cells and limits Flt3L-mediated anti-tumor immunity (88). Plasma cell-like pre-dendritic cells (pDCs) are a type of dendritic cell that plays a crucial role in antiviral immunity. Studies have demonstrated that increased densities of tumor-infiltrating pDCs are linked to longer survival rates in patients with colon cancer (89). In the tumor microenvironment, pDCs induce apoptosis in innate lymphoid cells ILC3s through the CD95 pathway (90). The study found that Bone marrow-derived dendritic cell (BMDC) H3K79me2 up-regulates forkhead box transcription factor M1 (FOXM1) in colorectal cancer by inhibiting BMDC maturation phenotype and function through the Wnt5a signaling pathway leading to anti-tumor effects (91). SUMO-specific protease 3 (SENP3) senses oxidative stress to promote STING-dependent dendritic cell antitumor function (92).

Interactions between dendritic cells (DCs) and other cellular components within the tumor microenvironment play a pivotal role in driving tumor progression. Cancer-associated fibroblasts (CAFs) secrete WNT2, which exerts inhibitory effects on DC-mediated anti-tumor T-cell responses by suppressing the SOCS3/p-JAK2/p-STAT3 signaling pathway, thereby facilitating immune evasion by tumors (93). Extracellular vesicles (EVs) secreted by tumors that contain miR-424 have been found to inhibit the CD28-CD80/86 co-stimulatory pathway in tumor-infiltrating T cells and dendritic cells. This leads to resistance to immune checkpoint blockade. Additionally, dendritic cells that are triggered by exosomes rich in miR-155 have been found to inhibit the growth of colorectal cancer tumors (8). Most studies have found that the expression of programmed cell death protein ligand 1 (PD-L1) on T cells is associated with tumor progression. However, a recent study has shown that the expression of PD-L1 on dendritic cells (DCs) is associated with improved survival in stage III colon cancer (94).

### 3.5 Myeloid-derived suppressor cells

Myeloid-derived suppressor cells (MDSCs) are a component of the tumor microenvironment (TME) and possess strong immunosuppressive capabilities. They modulate the immune response to cancer by expressing various protein molecules that interact with other molecules in the TME, thereby regulating colorectal carcinogenesis. MDSCs express a receptor for netrin-1, and netrin-1 enhances the immunosuppressive function of MDSCs through adenosine receptor 2B (A2BR), thereby promoting the development of colorectal cancer (9). Non-steroidal inflammatory drugs have a role in preventing colorectal cancer. Dectin-1 is a receptor for beta-glucan that plays a crucial role in host defense against fungi and in maintaining intestinal immune homeostasis. Blocking Dectin-1 can prevent colorectal cancer by inhibiting prostaglandin E2 production and enhancing the expression of interleukin-22 (IL-22)-binding proteins in myeloid-derived suppressor cells (95). Deletion of protease-activated receptor 2 (PAR2) in MDSCs promotes the progression of colorectal cancer by facilitating the production of reactive oxygen species mediated by transcription activator 3 (STAT3) (96). MDSC produces interleukin-10 (IL-10), which activates STAT3 to upregulate DNMT3b. This, in turn, silences the oncogene IRF8 in colonic epithelial cells, promoting colitis-associated colon tumorigenesis (97). The production of nitric oxide by MDSCs results in a decrease in interferon (IFN) responsiveness in immune cells (98). The STAT3 signaling pathway facilitates the accumulation of MDSCs, promoting colorectal cancer growth. G-CSF is implicated in this process (99). Inhibition of colorectal cancer growth can be achieved by inhibiting tumor neoangiogenesis and myeloid-derived suppressor cell aggregation through the use of reticulin 1 (ITLN1) (100). The junction protein CARD9 limits myeloid-derived suppressor cell expansion mediated by mycobacterial colonies, thereby inhibiting colon cancer (101). The tumor immunity suppression caused by GSK126, an inhibitor of histone methyltransferase EZH2, is due to its promotion of myeloid-derived suppressor cell production (102). S100A9 regulates the immunosuppression mediated by MDSCs through the RAGE and TLR4 signaling pathways in colorectal cancer (103). Fructose 1,6-bisphosphatase 1 (FBP1) inhibits colorectal tumorigenesis by suppressing the NF-κB pathway and mobilization of MDSCs (104).

Research indicates that myeloid-derived suppressor cells expressing chemokine receptor 2 (CXCR2) play a crucial role in promoting colitis-associated tumorigenesis (105). The promotion of colorectal cancer by Chemokine (CCL2) occurs through the enhancement of polymorphonuclear (PMN)-MDSC immunosuppressive features via the STAT3 pathway (66). The marker for gastrointestinal cancer stem cells, dicortin-like kinin (DCLK1), promotes the growth of colorectal cancer by inhibiting the function of tumor-specific cytotoxic T-lymphocytes through the recruitment of myeloid-derived suppressor cells (MDSCs) via the CXCL1-CXCR2 axis (106). Colorectal cancer progression is promoted by SMAD4 deficiency, which leads to the accumulation of MDSCs through the CCL15-CCR1 chemokine axis (107).

Additionally, other cells or molecules in the tumor microenvironment interact with myeloid-derived suppressor cells, which can influence colorectal cancer cell progression. Studies have shown that γδT17 cell infiltration positively correlates with colorectal cancer tumor stage and other clinicopathological features. γδT17 cells promote the accumulation and expansion of myeloid-derived suppressor cells in human colorectal cancer, which in turn promotes colorectal cancer growth (108). The activity of bone marrow-derived suppressor cells is promoted by mast cells, which contribute to the development of a microenvironment that is favorable to tumor growth (109). MDSCs promote tumor growth by suppressing CD8+ T cell cytotoxic activity (105). AlkB homologue 5 (ALKBH5) induces accumulation of MDSCs but reduces natural killer cells and cytotoxic CD8+ T cells, which in turn promotes colorectal carcinogenesis (110). Plasma cell-like dendritic cells (pDCs) can reduce colorectal cancer by modulating infiltration of myeloid-derived suppressor cells (111). The signaling of protein kinase 3 (RIPK3) in MDSCs promotes intestinal tumors by increasing tumor size. This is achieved through the expansion of interleukin-17 (IL-17)-producing T cells in MC38 tumors (112). Toll-like receptor agonists (TLR 7/8) reverse oxaliplatin resistance in colorectal cancer by directing myeloid-derived suppressor cells to tumor-killing M1 macrophages (113).

Toll-like receptor agonists (TLR 7/8) can reverse oxaliplatin resistance in colorectal cancer by directing myeloid-derived suppressor cells to tumor-killing M1 macrophages. Several drugs that act on MDSCs have been shown to clinically affect colorectal cancer development. Metformin, for example, activates the AMPK/mTOR pathway, leading to down-regulation of the mevalonate pathway, which reduces M2 macrophages and MDSCs and inhibits colorectal cancer development (114). Hydroxycumaric acid (MA) reduces the risk of colitis-associated colorectal cancer by inhibiting the recruitment of MDSCs through the suppression of IL-17 expression in γδT17 cells (115). Bojungikki-Tang (BJIKT) is a traditional Chinese herbal formulation used in Chinese medicine. When combined with anti-PD-L1 treatment, it significantly inhibits tumor growth, increases the proportion of cytotoxic T-lymphocytes and natural killer cells in tumor tissues, and suppresses the number of MDSCs in MC38-loaded mice (116).

MDSCs are associated with multiple metabolic pathways in relation to colorectal cancer. They produce nitric oxide which can lead to reduced interferon (IFN) responsiveness in immune cells (98). In human colorectal cancer, MDSCs promote tumor progression by inhibiting nitric oxide (NO) and reactive oxygen species (ROS) production through oxidative metabolism (117). Candida tropicalis fungus promotes colorectal carcinogenesis by inducing NLRP3 inflammasome activation through myeloid-derived inhibition of glycogen metabolism-dependent glycolysis and the JAK-STAT1 signaling pathway in cells (118) (**Table 1**).

**Table 1:** Functional mechanisms of innate immune cells in CRC.

		Related Pathways	fiction	Acts with colorectal cancer cells	Related articles
Tumor-associated macrophages	miR-195-5	Regulating the NOTCH signalling pathway	Induction of M2-like polarisation in macrophages	faciliating grow	(11)
	circVCP	Regulating the miR-9-5p/NRP1 signalling pathway	Induction of M2-like polarisation in macrophages	faciliating grow	(12)
	miR-186-5p	Regulating the DLC1 signalling pathway	Induction of M2-like polarisation in macrophages	faciliating grow	(13)
	Wnt5a	Regulating the CaKMII-ERK1/2-STAT3 signalling pathway	Induction of M2-like polarisation in macrophages	faciliating grow	(19)
	TCF4	Regulating the CCL2/CCR2 signalling pathway	Regulation of tumour-associated macrophages	faciliating grow	(22)
	up-regulation CXCL11		Enhanced expression of transforming growth factor in tumour-associated macrophages	faciliating grow	(25)
	MFHAS1	Activates STAT6 and KLF	Induction of M2-like polarisation in macrophages	faciliating grow	(34)
	TAMs	Induced SAA1		faciliating grow	(36)
	CXCL8		Induction of M2-like polarisation in macrophages and inhibition of CD8T cell infiltration	faciliating grow	(39)
	CCL5	Regulating the p65/STAT3-CSN5-PD-L1 signalling pathway		facilitating immune escape	(40)
	Agpat4/LPA	Regulating the p38/p65 signalling pathway	Induction of M1-like polarisation in macrophages and promotes T-cell activation	faciliating grow	(42)
	ILC1	Secretion of IFN-γ	Induction of M1-like polarisation in macrophages	faciliating grow	(45)
Neutrophil	IL-8	Regulating the GLUT3/GFAT	Induced modifications of O-GlcNAc	Regulating CSC-like properties of colon cancer cells	(51)
	up-regulation SPTAN1	Regulating the IL-8	Recruitment of neutrophils	faciliating grow	(53)
	HIF-2α	Regulating the CXCL1-CXCR2 signalling pathway	Recruitment of neutrophils	faciliating grow	(54)
	up-regulation SMAD4	Regulating the CXCL1/8-CXCR2 signalling pathway	Recruitment of neutrophils	faciliating grow	(55)
	CXCL1/CXCL2	Regulating the IL-1βsignalling pathway	Recruitment of neutrophils	faciliating grow	(56)
	PGE2	Regulating the E2-EP2 signalling pathway		faciliating grow	(57)
	AGR2			faciliating grow	(59)
	Neutrophil	Regulating the miR-155	down-regulation RAD51	inhibition grow	(64)
	CCL2	increase PMN-MDSCs		faciliating grow	(66)
	Neutrophil	Activation of TGFβ by MMP		inhibition grow	(67)
	SAA1		Recruitment of neutrophils	faciliating grow	(68)
	MMP-9/VEGF/HGF			faciliating grow	(69)
Natural killer cell	IL-15			Promotes growth and activation of cytotoxic CD8T and NK cells	(73)
	mSCs	regulating CXCL9	Promotes NK cell proliferation	Causes anti-tumour immunity	(75)
	NLRP3	Regulating theIL-18 signalling pathway	Influence on maturation of hepatic NK cells, surface expression of the death ligand FasL	inhibition grow	(76)
	blackberry		by enhancing Smad4 expression in NK cells	inhibition grow	(77)
	STING	Regulating the4-1BBL/4-1BB signalling pathway	Promotes the production of NK cell	inhibition grow	(82)
Dendritic cell	MLKL	Regulating theMEK/ERK signalling pathway	Suppression of the inflammatory response	inhibition grow	(86)
	TADCs		High expression of CCL5	faciliating grow	(87)
	WNT2	inhibiting the SOCS3/p-JAK2/p-STAT3 signalling pathway	Inhibition of DC-mediated anti-tumour T-cell responses	facilitating immune escape	(93)
Myeloid-derived suppressor cells	netrin-1	Regulating the A2BR signalling pathway	Enhancement of immunosuppression in MDSCs	faciliating grow	(9)
	down-regulation Dectin-1	Enhanced expression of IL-22 binding protein	Inhibition of prostaglandin E2 production in MDSC	inhibition grow	(95)
	down-regulation PAR2		Promotion of STAT3-mediated reactive oxygen species production	faciliating grow	(96)
	IL-10	Activation of STAT3 upregulates DNMT3b	Silencing of oncogene IRF8 expression	faciliating grow	(97)
	G-CSF	Regulating the STAT3 signalling pathway	Promotes the production of MDSC	faciliating grow	(99)
	ITLN1/CARD9/pDCs		Inhibition of MDSCs mobilisation	inhibition grow	(100, 101, 111)	
	GSK126/γδT17/ALKBH5		Promotes the production of MDSCs	faciliating grow	(102, 108, 110)	
	S100A9	Regulating the RAGE/TLR4 signalling pathway	Promotes the production of MDSCs	faciliating grow	(103)
	FBP1	inhibiting the NF-κB signalling pathway	Inhibition of MDSCs mobilisation	inhibition grow	(104)
	DCLK1	Regulating the CXCL1-CXCR2 signalling pathway	Promotes the production of MDSCs	faciliating grow	(106)
	down-regulation SMAD4	Regulating the CCL15-CCR1 signalling pathway	Promotes the production of MDSCs	faciliating grow	(107)
	RIPK3		Increasing tumour size by expansion of IL-17-producing T cells	faciliating grow	(112)
	MA	Inhibition of IL-17 expression in γδT17 cells	Inhibition of MDSCs mobilisation	inhibition grow	(115)
	metformin	Activation of the AMPK/ mTOR pathway leads to downregulation of the mevalonate pathway	Inhibition of MDSCs mobilisation	inhibition grow	(114)
	Fungus Candida tropicalis	Regulating the JAK-STAT1 signalling pathway	Induction of NLRP3 inflammatory vesicle activation	faciliating grow	(118)

## 4 Colorectal cancer immunotherapy

**Figure 2:**
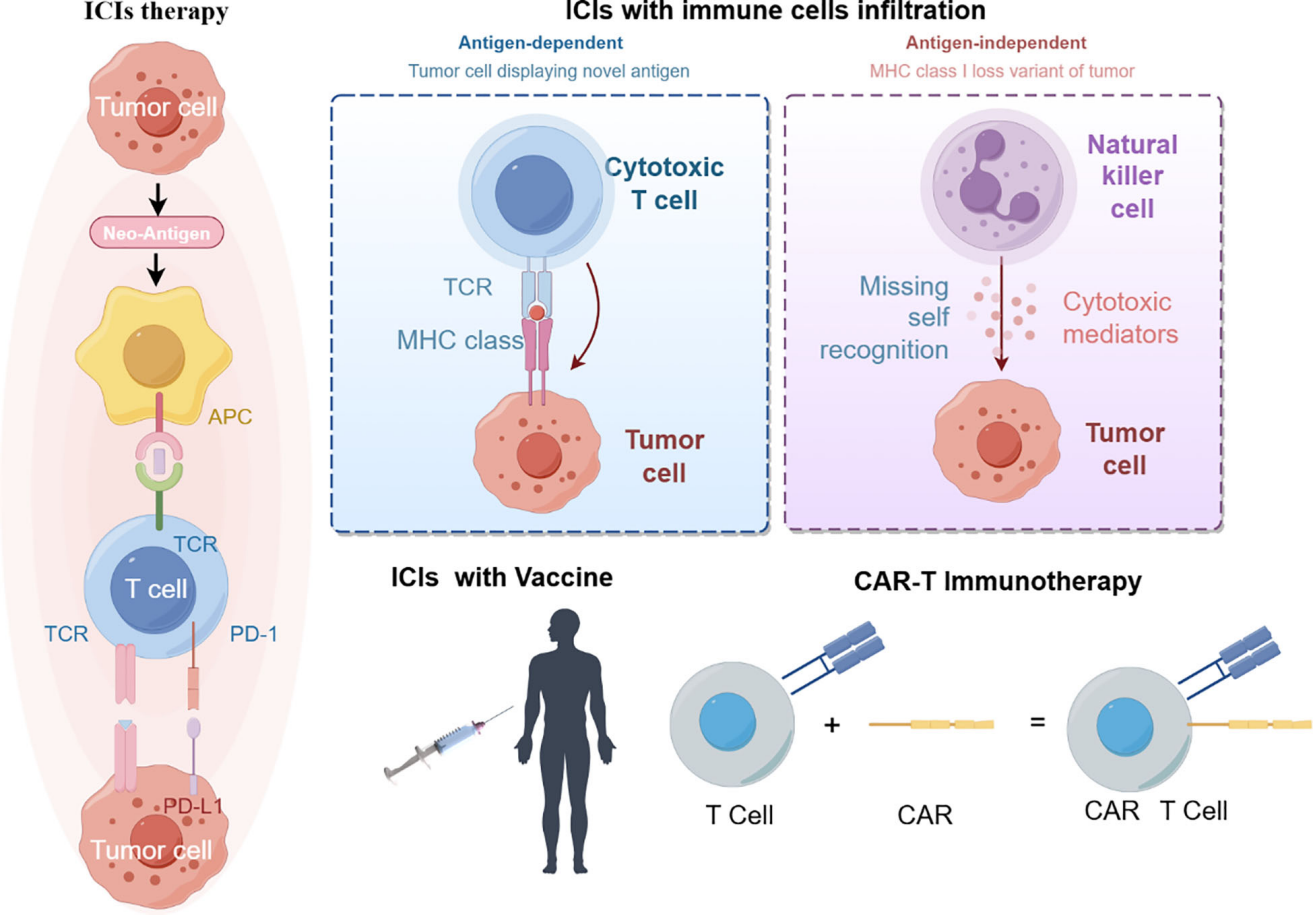
immunotherapy treatment (By figdraw).

Current immunotherapies have improved the survival of some colorectal cancer patients. Anti-tumor immunity requires organized and spatially subtle interactions between the components of the tumor immune microenvironment (**Figure 2**).

Immunotherapy for macrophages is currently under investigation using immune checkpoint inhibitors, monoclonal antibodies, cellular relay therapies, and combination therapies. The aim is to directly reduce the number of tumor-associated macrophages (TAMs), prevent their recruitment, enhance phagocytosis and killing, or target surface molecules of TAMs. Numerous studies have shown that TAMs are potential targets for immunotherapy of colorectal cancer (CRC) (119, 120). The study found that certain clinical drugs, such as metformin, Yunnan Garcinia Cambogia, and Mongolian Astragalus-Yulin, can decrease the recruitment of TAMs (114, 121, 122). The PD-1/PD-L1 immune checkpoint inhibitor enhances the phagocytosis of tumor-associated macrophages (TAM) and reduces tumor load (123). Several combination therapies are currently in progress. Liposomes (TAT-BLZmlips) with magnetic navigation and magnetic heat therapy (MHT) can repolarize M2, inhibiting tumor growth and recurrence (124). The combination of cryophotothermal therapy (PTT) and anti-PD-L1 immunotherapy has been reported to improve efficacy against colorectal cancer (125). Combined furazinotinib chemotherapy and anti-PD-1 therapy promotes an anti-tumor immune response (123).

Neutrophils are a cellular target of immunotherapy. Current research on tumor-associated neutrophils (TANs) aims to reduce their numbers and inhibit their accumulation in tumors. Studies have shown that increased accumulation of TANs in tumors hinders the response to anti-PD-1 drug therapy (126). The combination of oxaliplatin chemotherapy and immunotherapy has been found to enhance lipid A immunotherapy efficacy by recruiting neutrophils (127). Further research is needed to develop other immunotherapies targeting neutrophils.

In recent years, there has been an increase in research on NK cell immunotherapy. However, there has been limited research on its effectiveness in treating colorectal cancer. One way to enhance the activity of NK cells and improve their ability to kill tumors is through cytokine supplementation. For instance, the survival time of colorectal cancer patients can be prolonged by supplementing with IL-12 (74). Additionally, NK cells have been utilized in genetic engineering. The CAR-NK cells, which are genetically engineered immune cells equipped with chimeric antigen receptors (CARs) for precise targeting of tumor-specific antigens, have exhibited promising outcomes in preclinical investigations focusing on colorectal cancer (128).

Dendritic cells play a crucial role in anti-tumor immunity. Relevant immunotherapies include *in vivo* activation of DCs, chemotherapy combination therapy, *in vivo* expansion, and DC vaccines. Clotrimazole can activate DCs through the lactate-lysosome axis, promote antigen cross-presentation, and enhance the effect of anti-PD-1 immunotherapy (129). The use of immune adjuvant nanocomplexes and dendritic vaccines, in combination with immune checkpoint blockade, has been shown to be effective in cancer therapy (130). Electrothermal therapy and dendritic cell immunotherapy have been used to improve the immune-tumor microenvironment (131). Although initial results in glioblastoma and metastatic melanoma are promising, DC vaccines have not yet been fully explored in colorectal cancer (132).

Modifying the suppressive effects of myeloid-derived suppressor cells (MDSCs) is currently considered a promising cancer therapy in colorectal cancer. A study has shown that histamine-targeted depletion of MDSCs enhances the efficacy of PD-L1 blockade in colorectal cancer (133). Additionally, supramolecular tadalafil nanovaccines have been used in cancer therapy to attenuate MDSCs and enhance immunogenicity (134). Chemotherapeutic agents, such as paclitaxel, reduce tumor progression by inhibiting reactive oxygen species and nitric oxide produced by MDSC cells (135). Rhythmic therapy targeting poly ADP-ribose polymerase-1 (PARP-1) modulates MDSC inhibition and enhances anti-PD-1 immunotherapy in colon cancer (136). Antagonizing the signaling of the prostaglandin E2 axis effectively reduces recruitment and differentiation of MDSCs, thereby controlling tumor growth (95).

Immunotherapy is a therapeutic approach that aims to harness the immune system to fight cancer. As illustrated above, immune checkpoint inhibitors (ICIs) regulate the interactions between T cells, antigen-presenting cells (APCs) and tumor cells, thereby facilitating the release of suppressed immune responses. Immunotherapy is currently effective in patients with metastatic colorectal cancer (mCRC) who have defective mismatch repair (dMMR) or high microsatellite instability (MSI-H) (referred to as dMMR/MSI-H mCRC). Many clinical trials have also been conducted in patients with defective mismatch repair (pMMR) or microsatellite stabilization (MSS) or low microsatellite instability (MSI-L) (referred to as pMMR/MSI/MSI-L mCRC) in patients with mCRC (**Table 2**).

**Table 2:** Ongoing trials in dMMR-MSI-H CRC and pMMR-MSI-L CRC.

CRC	Clinicaltrials.gov Identifier	Phase	Checkpoint inhibitors	Primary endpoint
dMMR-MSI-H	NCT02563002	III	Pembrolizumab versus standard-of-care chemotherapy	PFS, OS, ORR
	NCT02060188	II	Nivolumab ± ipilimumab or daratumumab or anti-LAG3 antibody	ORR
	NCT03026140	II	Nivolumab + ipilimumab ± celecoxib	Safety
			
	NCT01876511	II	Pembrolizumab	ORR
	NCT02060188	II	Nivolumab ± ipilimumab	PFS, OS, ORR
pMMR-MSI-L	NCT02888743	II	Duvalumab (PD-1) + Tremelimumab (CTLA-4)	ORR
	NCT03007407	II	Duvalumab (PD-1) + Tremelimumab (CTLA-4)	ORR
	NCT03104439	II	Nivolumab (PD-1) + Ipilimumab (CTLA-4)	DCR
	NCT04030260	II	Nivolumab (PD-1)±Regorafenib (Multikinase)	PFS
	NCT02060188	II	Nivolumab (PD-1) ± Ipilimumab (CTLA-4)+Cobimetinib (MEK)	ORR
	NCT02788279	III	Atezolizumab (PD-L1)+Cobimetinib (MEK) + Regorafenib (multikinase)	OS, PFS, OR
	NCT01633970	I	Atezolizumab (PD-L1)+Bevacizumab (VEGF)	AEs, DLTs, MTD

PFS, progression-free survival; OS, overall survival; ORR, overall response rate; PD-1, programmed cell death 1; CTLA-4, cytotoxic T-lymphocyte-associated protein 4; DCR, disease control rate; DLTs, dose-limiting toxicities; AEs, number of adverse events; SAEs, number of serious adverse events; PD-L1, programmed cell death-Ligand1; MTD, maximum tolerated dose

## 5 Conclusions and outlook

In the tumor microenvironment, innate immune cells play a crucial role in the development of colorectal cancer. Depending on the microenvironment and stimuli, they can either promote or suppress the growth of colorectal cancer. Innate immune cells can promote inflammation, angiogenesis, drug resistance, and cancer growth by secreting certain substances or undergoing changes in phenotype. Tumor development is closely linked to the reconfiguration of cellular metabolic processes. A hallmark of cancerous cells’ metabolism is their capacity to secure vital nutrients from environments that are typically nutrient-deficient. They utilize these nutrients not only to sustain their survival but also to produce additional cellular material. Changes in both the internal and external metabolic substances can occur alongside the metabolic reprogramming associated with cancer, significantly influencing gene expression, cell differentiation, and the surrounding environment of the tumor. Although the current treatment for colorectal cancer (CRC) patients is primarily based on surgery, radiotherapy, and chemotherapy, immunotherapy is gradually being introduced as a treatment option. Therefore, it is essential to have a comprehensive understanding of how innate immune cells and related molecular mechanisms impact the development of CRC. Current immunotherapy has significantly improved the survival rate of colorectal cancer patients. However, it also has several limitations, including immune adverse reactions and enhanced drug resistance. Therefore, in the future, we should explore the mechanism of immune cells on colorectal cancerIsekai Cheat Magician development, search for more accurate tumor markers, and investigate new methods of combination therapy. This will enable us to formulate more effective and individualized therapeutic strategies.

## Author contributions

WD: Writing – review & editing. WL: Writing – original draft. TK: Writing – original draft. LL: Writing – original draft.
